# Strengthening preventive care programs: a permanent challenge for healthcare systems; lessons from PREVENIMSS México

**DOI:** 10.1186/1471-2458-10-417

**Published:** 2010-07-14

**Authors:** Gonzalo Gutiérrez, Ricardo Pérez-Cuevas, Santiago Levy, Hortensia Reyes, Benjamín Acosta, Sonia Fernández Cantón, Onofre Muñoz

**Affiliations:** 1 Hospital Infantil de México "Federico Gomez". Dr. Márquez 162, Colonia Doctores, México DF., (Postal code 06720), México; 2 Unidad de Investigación Epidemiológica y en Servicios de Salud CMN Siglo XXI, Instituto Mexicano del Seguro Social. Avenida Cuauhtémoc 330, Colonia Doctores, México DF., (Postal code 06720), México; 3 Inter-American Development Bank. Stop B-900, Washington DC., (Postal code 20577), USA; 4 Centro de Investigación en Sistemas de Salud. Instituto Nacional de Salud Pública. Avenida Universidad 655, Colonia Santa Maria Ahuacatitlán, Cuernavaca, Morelos, (Postal code 62508), México; 5 Unidad de Salud Pública, Instituto Mexicano del Seguro Social. Mier y Pesado 20, Colonia del Valle, Delegación Benito Juarez, México DF., (Postal code 03100), México; 6 Subsecretaria de Promoción y Prevención a la Salud. Secretaría de Salud. Francisco P. Miranda 77, Colonia Merced Gómez, Delegación Alvaro Obregón, México DF., (Postal code 01600), México

## Abstract

**Background:**

In 2001, the Instituto Mexicano del Seguro Social (IMSS) carried out a major reorganization to provide comprehensive preventive care to reinforce primary care services through the PREVENIMSS program. This program divides the population into programmatic age groups that receive specific preventive services: children (0-**9 **years), adolescents (**10**-**19 **years), men (20-59 years), women (20-59 years) and older adults (> = 60 years). The objective of this paper is to describe the improvement of the PREVENIMSS program in terms of the increase of coverage of preventive actions and the identification of unmet needs of unsolved and emergent health problems.

**Methods:**

From 2003 to 2006, four nation-wide cross-sectional probabilistic population based surveys were conducted using a four stage sampling design. Thirty thousand households were visited in each survey. The number of IMSS members interviewed ranged from 79,797 respondents in 2003 to 117,036 respondents in 2006.

**Results:**

The four surveys showed a substantial increase in coverage indicators for each age group: children, completed schemes of vaccination (> 90%), iron supplementation (17.8% to 65.5%), newborn screening for metabolic disorders (60.3% to 81.6%). Adolescents, measles - rubella vaccine (52.4% to 71.4%), hepatitis vaccine (9.3% to 46.2%), use of condoms (17.9% to 59.9%). Women, measles-rubella vaccine (28.5% to 59-2%), cervical cancer screening (66.7% to 75%), breast cancer screening (> 2.1%). Men, type 2 diabetes screening (38.6% to 57.8%) hypertension screening (48-4% to 64.0%). Older adults, pneumococcal vaccine (13.2% to 24.9%), influenza vaccine (12.6% to 52.9) Regarding the unmet needs, the prevalence of anemia in children was 30% and a growing prevalence of overweight and obesity, type 2 diabetes, and hypertension was found in men, women and older adults.

**Conclusion:**

PREVENIMSS showed an important increase in the coverage of preventive services and stressed the magnitude of the old and new challenges that this healthcare system faces. The unsolved problems such as anemia, and the emerging ones such as overweight, obesity, among others, point out the need to strength preventive care through designing and implementing innovative programs aimed to attain effective coverage for those conditions in which prevention obtains substandard results.

## Background

It has been a long-standing fact that curative care receives most of the healthcare budgets [1]; however, preventive care is receiving further attention from scholars, politicians and decision makers given its effectiveness on people's health and its long-term effect on social expectancies and well-being [2] Current emphasis has shifted toward cost-effective delivery of healthcare [3] which implies finding equilibrium between curative and preventive care through reinforcing primary care services [4]. The World Health Organization is a strong advocate to renew primary health care (PHC) pointing out that it is the cornerstone of health systems and is the best way to provide comprehensive, equitable and affordable health care. Preventive care is within the main components of PHC and when the provision is comprehensive, it increases the access and uptake of preventive services, which in turn contributes to obtain better health and improved quality [5] Providing preventive services within PHC facilitate to obtain both, technical and productive efficiency. A number of technical documents have stressed the importance of prioritizing health interventions to better allocate the scarce resources [6-10].

The Instituto Mexicano del Seguro Social (IMSS) is the largest public healthcare system in Mexico. It is a nationwide institution that administratively is divided in state delegations. IMSS provides social, economic and health protection to workers of the formal sector and their families. The workers of the formal sector are those employed with regular wages and hours, with employment rights and tax payments. Its members work in the industry and in the services sector such as commerce, transportation, etc. [11] IMSS provides services in urban areas and almost all of its members have basic sanitary conditions (water, electricity, sewerage, etc.).

Healthcare benefits comprise preventive, curative and rehabilitation care that is provided in primary care clinics, secondary and tertiary care hospitals. IMSS revenues come from three parties: the government, the employers and the employees. The latter pay the premium according to their income.

Currently, IMSS provides care to approximately 48 million members. Since the year 2000, this institution reoriented its vision regarding the provision of medical care and began to search for an appropriate balance in its healthcare expenditures for both curative and preventive care. In 2007, chronic conditions such as type 2 diabetes, hypertension, chronic renal failure, cervical cancer, breast cancer and HIV/AIDS accounted for 12.15% of the total IMSS healthcare expenditures. The projections for the year 2050, using an optimistic scenario that includes the strengthening of preventive measures and technological innovation, estimated that the percentage of IMSS healthcare expenditures for these seven conditions would be 22% and the pessimistic scenario (not investing in preventive and curative care) estimated an increase of 57% in health expenditures [11].

To strengthen preventive care, IMSS carried out a situation analysis of the way in which these services were provided. The analysis showed: 1) lack of coordination to provide preventive care. There were > 30 isolated preventive programs (i.e., vaccination program, family planning program, cervical cancer screening program, and so on). These programs were competing among themselves for resources and personnel; 2) gaps in the health information system that was unable to provide exact figures regarding its coverage. To tackle these flaws, the institution developed the program PREVENIMSS (the Spanish acronym for IMSS' Integrated Preventive Care Program) that aimed at improving the delivery of service, and at evaluating the progress of coverage of preventive care services. The usual definition of coverage is the regular update of the proportion of individuals who need an intervention and actually receive it; therefore, information about coverage is key to evaluate health programs.

Three strategies integrated the organizational changes supporting the implementation of this program: (1) Integration of the scattered preventive activities into a comprehensive package. (2) Reorientation of evaluation criteria, shifting from evaluation of productivity to evaluation of coverage. IMSS launched PREVENIMSS in 2001 and this was accompanied by a permanent mass media campaign with radio and television advertisements. A careful description of PREVENIMSS has been published elsewhere [12].

1. Integration of preventive services. PREVENIMSS reorganized the provision of preventive services by programmatic age groups: children (0-9 years), adolescents (10-19 years), women (20-59 years), men (20-59 years) and older adults (60 years and older). A number of organizational and procedural changes took place at central, district and local level. The old appointment booklets for IMSS individual members were redesigned to include preventive information, dated registries of preventive services, and reminders tailored to suit each programmatic age group. The booklet is the official document where preventive services are being registered each time that an IMSS member receives preventive care at IMSS facilities. Thus, the booklet contains registries about vaccines, screening and educational activities. It also registers the appointments to provide programmed preventive services. Each IMSS member has his/her individual booklet.

Preventive services for each age group were reviewed and updated continuously. Table 1 shows the preventive services for each age group. The broad areas of preventive services were: health promotion, nutrition, prevention, control and screening of selected diseases.

**Table 1:** PREVENIMSS main activities by age group

Activities	Children	Adolescents	Women	Men	Older adults
	0-10 years	11 -19 years	20-59 years	20-59 years	> = 60 years
**Health promotion**	**Delivery of PREVENIMSS booklets**				

	**Measurement of height, weight and waist**				
	
Nutrition	Iron supplementation Vitamin A supplementation Intestinal parasites treatment	Intestinal parasites treatment Folic acid supplementation (pregnant teenagers)	Detection of anemia; iron supplementation; folic acid supplementation (pregnant women)		

Prevention and control of diseases	Vaccines: BCG, Sabin; DPT+HB+Hlb; Influenza; measles, rubella, pertussis, Oral rehydration therapy for acute diarrhea, identification of alarm signs in acute respiratory infections	Vaccines: measles-rubella, tetanus toxoid, **two-dose **hepatitis B, Provision of condoms to prevent STDs and HIV/AIDS and unwanted pregnancies	Vaccines: measles-rubella, tetanus toxoid, diphtheria Tuberculosis: screening and directly observed treatment	Vaccines: measles-rubella, tetanus toxoid. Tuberculosis: screening and directly observed treatment	Vaccines: pneumonia, influenza, tetanus toxoid and diphtheria; Tuberculosis: screening and directly observed treatment

Screening	Congenital hypothyroidism, Phenylketonuria. Congenital adrenal hyperplasia, Biotinidase deficiency, Visual acuity, Childhood caries	Visual acuity	Cervical cancer Breast cancer Type 2 diabetes Hypertension	Type 2 diabetes Hypertension	Cervical cancer Breast cancer Type 2 diabetes Hypertension

Reproductive health		Family planning and antenatal care		Family planning	

2. Reorientation of evaluation criteria. The former criterion to evaluate the progress of preventive actions was productivity; the criteria were reoriented to evaluate coverage. The registries of productivity served only to ascertain the number of preventive actions provided; no denominator was used for this measure. Instead, for coverage, criteria to receive a preventive action were defined to meet the health needs of the affiliated population; i.e., immunization schemes according to age and dose, or periodicity of cervical cancer screening based upon risk factors. This decision helped to focus the provision of preventive actions based in actual health needs rather than in the percentage of people receiving preventive services.

With the aim of showing the complexity of implementing large scale preventive care programs to reinforce PHC, the objective of this paper is to describe the increase of coverage of preventive actions through the PREVENIMSS program and the magnitude of the unmet needs of some of the most important unsolved and emergent health problems, such as anemia in children, and the growing increase in the prevalence of overweight and obesity and of its consequences, type 2 diabetes and hypertension.

## Methods

The evaluation of PREVENIMSS' coverage was conducted through four population surveys that were carried out in the years 2003, 2004, 2005 and 2006. These surveys were called ENCOPREVENIMSS for its Spanish acronym: PREVENIMSS National Coverage Surveys. All four nation-wide cross sectional surveys were designed as probabilistic, population-based. The study population was all IMSS members across the country. IMSS considers as a member a person who is entitled to receive social security services within which healthcare is included; this comprises the insured and their beneficiaries (spouse, children, and parents).

The information of preventive care was obtained through home interviews and included all IMSS members living in the house, whether or not they had used IMSS services or looked for care in other healthcare institutions, either public or private. The answers provided by the interviewee were confirmed by reviewing the information registered at the PREVENIMSS booklet.

The surveys had ethical approval from the IMSS Institutional Review Board. All participants received information about the purpose of the study and were asked for their informed consent before starting the interview. To collect information from children, the mother or caretaker should have to provide her informed consent.

### Sampling design

The sampling design took into account that IMSS is divided into 37 state delegations. The surveys were planned to be representative in every state delegation for each programmatic age group. A four-stage sampling design was used. In the first stage, six family medicine clinics all belonging to the IMSS health care system were randomly chosen at each state delegation; this represented a total of 222 family medicine clinics. At the second stage, the geographic area of influence of each family medicine clinic was considered; then, a portion of this area was randomly chosen. At the third stage, a specific neighborhood was randomly selected. The fourth stage consisted in identifying the households where IMSS members were living; the interviewers did home visits looking for IMSS members. The interviewers were up to three times to the house to contact the potential participant. If the interviewers were unable to contact the residents of the selected household or if they refused to participate, then, the household was replaced with another with similar characteristics.

The primary sampling unit was the household and the elementary unit was the IMSS member. We interviewed all household members entitled to receive IMSS services. This is because IMSS policy consists in providing health care to the worker and his/her family dependants.

To get the estimates of coverage per programmatic age group, the sample size for the surveys was calculated using the following formula:

$$\text{n}=\text{pq}\left[\frac{{\left(\text{Z}\propto /2\right)}^{2}}{\delta^{2}}\right]\frac{\text{DEFT}}{1-\text{NR}}$$

Assumptions: n = sample size, p = proportion of coverage (0.6), q = 1-p, α = 0.05, δ = 0.05, design effect (DEFT) = 1.2, and non-response rate (NR) = 10%.

In the first survey (2003) the proportion of coverage (p) was estimated to be 0.8. Thus the n for this survey was lower than for the surveys of the years 2004, 2005 and 2006.

The resulting sample size was 492 participants in each programmatic age group per state delegation. The total sample size per delegation was 2,460, which multiplied by the 37 state delegations resulted in ~91,000 individuals in each survey.

Sources of information and main variables

The PREVENIMSS booklet was the main source of information and as mentioned earlier, physical measures were taken in a subsample of interviewees for the 2006 survey.

The main variables in each programmatic age group were:

Children (0-9 years): registry of height and weight, iron supplementation, oral health activities, visual acuity measurement and vaccines scheme.

Adolescents: (10-19 years): registry of height and weight, oral health activities, visual acuity measurement, vaccines scheme and use of condoms.

Women (20-59 years): registry of height, weight and waist, screening for tuberculosis, cervical cancer, breast cancer, type 2 diabetes and hypertension.

Men (20-59 years): registry of height, weight and waist, screening for tuberculosis, type 2 diabetes and hypertension.

Older adults (60 years and older): registry of height, weight and waist, pneumococcal vaccine, influenza vaccine, screening of tuberculosis, cervical cancer (women), breast cancer (women), type 2 diabetes and hypertension.

Sociodemographic variables: age, sex, place of residence, literacy of individuals 5 years and older, occupation and size of the family (defined as the number of people living in the house).

In the 2006 survey, to complement the information, we took physical measures to estimate the prevalence of several conditions (unmet health needs). To obtain the information to estimate the prevalence of malnutrition, overweight and obesity, the interviewers measured height, weight, waist and hip circumferences to 25% of all interviewees. The interviewers were nurses that were previously trained and standardized to measure weight and height. All were independent from IMSS and hired for this survey.

The levels of cholesterol and blood glucose were measured in 25% of interviewees that were above 19 years old. The Accutrend^® ^GCT, Roche was used for this purpose.

The levels of hemoglobin to ascertain anemia were measured in 25% of children below 5 years. We used the B-hemoglobin photometer (HemoCue^®^, Ångelholm, Sweden) for this purpose.

Blood pressure measurements to 25% of interviewees older than 19 years were taken by using sphygmomanometers (TJX MD 3000).

Criteria to ascertain overweight and obesity were as follows:

• Children less than five years old: overweight, body mass index (BMI) between 2-3 Z score; obesity, > 3 Z score of WHO growth standard [13]

• Children 5 to 9 years: overweight and obesity BMI criteria of International Obesity Task Force [14]

• Adolescents: Overweight and obesity, BMI criteria of International Obesity Task Force [14]

• Women, men and older adults: overweight, BMI 25 to 29.9; obesity, BMI > = 30 [15]

Criteria for type 2 diabetes screening: fasting glucose ≥ 126 mg/dl; casual glucose ≥ 200 mg/dl.

Criteria for hypertension: systolic blood pressure ≥ 140 mm Hg in two subsequent measurements or diastolic blood pressure ≥ 90 mm Hg in two subsequent measurements in the same visit, at the beginning and at the end of the visit.

### Data analysis

The statistical analysis included the ascertainment of the proportion of IMSS members who received preventive services. This was evaluated according to each programmatic age group. The increase of coverage throughout the years was estimated by comparing the groups of subjects per age group, year and type of preventive care. The slopes were compared by running a simple regression analysis, and the assessment of the goodness of fit was done by calculating the correlation coefficients (r^2^) [16].

## Results

### Population Characteristics

The number of IMSS members interviewed ranged from 79,797 respondents in 2003 to 117,036 respondents in 2006. Table 2 shows the age distribution of the people interviewed. The age distribution corresponds fairly with the sample design and it should not be considered representative of the age distribution of IMSS members. The individual non-response rate in the four surveys was below 10%.

**Table 2:** Households and population respondents. ENCOPREVENIMSS 2003-2006

	2003		2004		2005		2006	
**Households with IMSS members**	**34,610**		**37,877**		**44,278**		**40,682**	

**Population respondents**	**No**.	**%**	**No**.	**%**	**No**.	**%**	**No**.	**%**

Children (< 10 years)	15,289	19.2	20,762	17.6	23,177	18.9	22,365	19.1
Teenagers (10 to 19 years)	13,356	16.7	20,259	17.2	21,474	17.6	20,701	17.7
Women (20 to 59 years)	22,165	27.8	30,910	26.2	32,317	26.4	29,939	25.6
Men (20 to 59 years)	16,275	20.4	25,745	21.8	25,375	20.7	24,507	20.9
Older adults (> 59 years)	12,712	15.9	20,208	17.1	20,037	16.4	19,524	16.7

Total	79,797	100.0	117,884	100.0	122,380	100.0	117,036	100%

### Coverage

#### Health programs for children

Coverage of preventive programs for children increased continuously: iron supplementation in children < 1 year (17.8% to 65.5%) prevention of childhood caries (40.5% to 58.1%) screening for congenital metabolic disorders phenylketonuria, congenital adrenal hyperplasia, biotinidase deficiency (60.3% to 81.6%) and visual acuity testing (12.5% to 47.5%) (table 3).

**Table 3:** Coverage of preventive services provided to children and adolescents

Coverage indicators in each age group	year				Linear slope	r^2 ^linear adjustment
			
	2003	2004	2005	2006		
**Children**	**n = 15,289**	**n = 20,762**	**n = 23,177**	**n = 22,365**		
	**%**	**%**	**%**	**%**	**%**	**%**

Delivery of PREVENIMSS booklets	32.1	62.7	77.2	90.7	19.0	95.5
Weight measurement	72.2	73.5	79.0	84.4		
Height measurement	56.6	70.3	76.0	81.7		
Iron supplementation in children < 1 year old	17.8	46.0	47.7	65.5		
Completed scheme of vaccination by age	91.4	91.0	91.4	90.3	-0.3	52.1
Fluoride application	40.5	42.0	43.2	58.1	5.4	72.7
Hypothyroidism screening	97.1	96.7	98.5	98.0	0.4	49.9
Screening for congenital adrenal hyperplasia, phenylketonuria and biotinidase deficiency	--	-	60.3	81.6	21.3	100.0
Visual acuity screening	12.5	22.5	32.1	47.5	11.5	98.6

**Adolescents**	**n = 13,356**	**n = 20,259**	**n = 21,474**	**n = 20,701**	**Linear slope**	**r**^**2 **^**linear adjustment**
	%	%	%	%	**%**	**%**

Delivery of PREVENIMSS booklets	25.9	54.6	68.9	84.3	19.0	97.0
Weight measurement	36.3	57.7	64.1	73.2	11.7	92.8
Height measurement	33.8	55.3	61.6	71.1	11.8	93.0
Measles-rubella vaccine	52.4	55.6	58.8	71.4	6.0	87.2
Tetanus toxoid and diphtheria vaccine	68.0	65.8	63.7	80.0	3.4	36.0
Hepatitis B vaccine	9.3	17.7	26.1	46.2	11.9	94.5
Use of condom in last intercourse	17.9	30.4	42.2	59.9	13.8	99.1
visual acuity screening	2.1	30.1	51.5	61.2	19.9	95.9

#### Health programs for adolescents

Almost all components of the adolescents program, excepting the vaccination program, were implemented right from the onset of PREVENIMSS. The activities included measurement of weight and height, vaccines: measles - rubella (52.4% to 71.4%), tetanus toxoid- diphtheria (68% to 80%) hepatitis (9.3% to 46.2%) There was also increase in the use of condoms (17.9% to 59.9%) and in visual acuity testing (2.1% to 61.2%), (Table 3).

#### Health programs for women

Measles-rubella vaccine increased from 28.5% to 59.2%, women undergoing cervical cancer screening for the first time or subsequent screening (three-year interval) increased from 66.7% to 75%. Breast cancer screening by using mastography began in 2004 and by the year 2006 its coverage was 22.1% (Table 4).

**Table 4:** Coverage of preventive services provided to women, men and older adults

Coverage indicators in each age group	Years				Linear slope	r^2 ^linear adjustment
						
Women	2003	2004	2005	2006		
	n = 22,165	n = 30,910	n = 32,317	n = 29,939		
	%	%	%	%	%	%
Delivery of PREVENIMSS booklets	34.9	66.5	80.5	90.0	17.9	92.5
Weight measurement	69.7	69.5	79.0	84.9	5.5	89.3
Height measurement	51.1	61.6	74.0	80.4	10.0	98.6
Waist measurement	8.1	17.2	26.4	52.9	14.4	91.9
Measles rubella vaccine	28.5	36.2	43.9	59.2	10.0	96.6
Breast cancer screening						
Clinical exam	42.6	45.3	50.4	62.4	6.5	90.2
Mastography		6.5	7.9	22.1	7.8	81.7
Cervical cancer screening						
Once in lifetime	81.3	78.8	82.4	86.9	2.0	60.5
Once in the last 3 years	66.7	72.4	74.5	75.0	2.7	84.0
Once in the last year	40.6	51.0	45.1	43.3	0.2	0.4
Diabetes mellitus screening	45.3	55.1	56.8	66.5	6.5	94.3
Hypertension screening	60.6	66.0	70.6	74.2	4.5	99.2

**Men**	**n = 16,275**	**n = 25,745**	**n = 25,375**	**n = 24,507**	**Linear slope**	**r**^**2 **^**linear adjustment**
	**%**	**%**	**%**	**%**	**%**	**%**

Delivery of PREVENIMSS booklets	25.1	55.3	70.9	85.1	19.6	96.3
Weight measurement	56.8	53.8	62	73.9	6.0	75.2
Height measurement	47.2	49.4	58.7	70.6	8.0	92.8
Waist measurement	3.8	9.7	16.6	45.1	13.1	85.2
Measles rubella vaccine	21.9	28.8	35.7	49.4	8.9	96.6
Diabetes mellitus screening	38.6	41.7	44.4	57.8	6.0	84.8
Hypertension screening	48.4	49.1	56.5	64.0	5.4	91.4

**Older adults**	**n = 12,712**	**n = 20,208**	**n = 20,037**	**n = 19,524**	**Linear slope**	**r**^**2 **^**linear adjustment**
	**%**	**%**	**%**	**%**	**%**	**%**

Delivery of PREVENIMSS booklets	49.3	75.5	84.4	92.7	13.9	91.1
Weight measurement	64.7	76.9	83.1	88.2	7.7	95.5
Height measurement	48.3	71.1	78	83.9	11.4	88.7
Waist measurement	4.5	13.7	23.5	52.9	15.5	90.9
Pneumococcal vaccine	13.2	24.4	23.6	24.9	3.4	63.1
Influenza vaccine	12.6	27	37.2	52.9	13.1	99.4
Tuberculosis vaccine	1.9	2.8	3.7	5.0	1.0	99.1
Diabetes mellitus screening	34.8	54.4	56	65.2	9.3	87.8
Hypertension screening	46.0	66.9	71.8	75.0	9.2	82.7

#### Health programs for men

Weight and height measurements increased (56.8% to 73.9% and 47.2% to 70.6% respectively), type 2 diabetes screening increased from 38.6% to 57.8% and hypertension screening increased from 48-4% to 64.0% (table 4).

#### Health programs for older adults

Pneumococcal vaccination coverage increased from 13.2% to 24.9%. Influenza vaccine coverage also increased from 12.6% to 52.9 (table 4).

The linear slopes and the r^2 ^linear adjustment outcomes show the strength of the linear relationship between PREVENIMSS and the increase in coverage for the different components of the program. This represents that the largest values in the table (closer to 1 or 100%) show the straight-line relationship between the program and the attained coverage figures.

### Unmet needs

Prevalence of anemia in children one to four years old (ENCOPREVENIMSS 2006): Figure 1 shows that children under one year of age had the highest prevalence (30.8%) of anemia. The level of incidence decreases progressively with age, the lowest figure being observed in children four years old (12%). The overall proportion of anemia in children 0-4 years was 19%.

**Figure 1:**
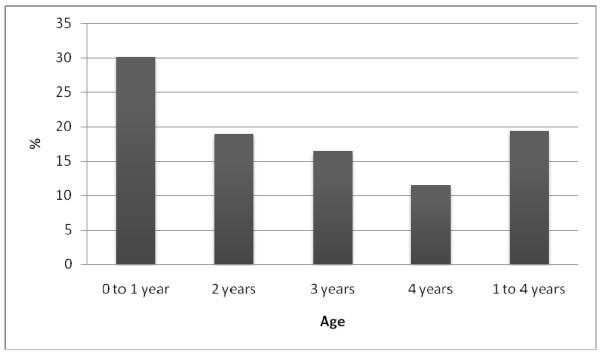
Prevalence of anemia in children from one to four years old*. *Only data from the 2006 EncoPrevenimss survey are showed in the figure. Source: Encoprevenimss 2006.

**Figure 2:**
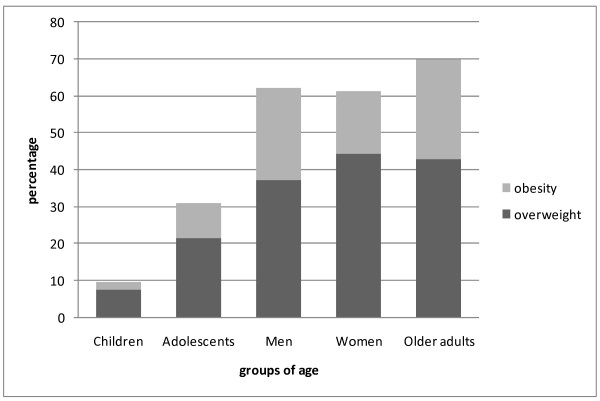
Prevalence of obesity and overweight in each age group*. *Only data from the 2006 EncoPrevenimss survey are showed in the figure. Source: Encoprevenimss 2006.

**Figure 3:**
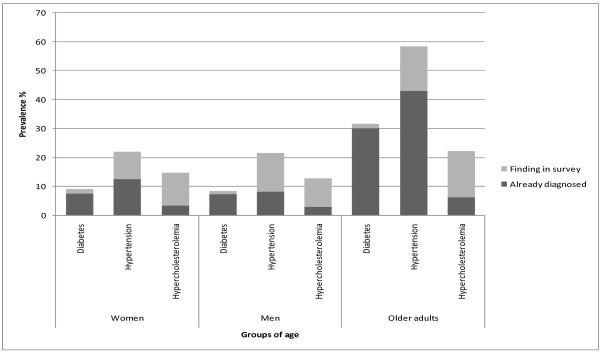
Prevalence of type 2 diabetes, hypertension and hypercholesterolemia: Patients already diagnosed and those identified in the survey*. *Only data from the 2006 EncoPrevenimss survey are showed in the figure. Source: Encoprevenimss 2006.

Overweight, obesity, type 2 diabetes and hypertension (Figures 2 and 3): Among the most important findings of ENCOPREVENIMSS are the prevalence of overweight and obesity, type 2 diabetes and hypertension, in both diagnosed and undiagnosed cases. The prevalence of overweight and obesity in every age group was as follows: children 9.5%; adolescents 30.9%; men 61.3%; women 62.1% and older adults 69.9%. The total prevalence of type 2 diabetes was 14.8%, and 10% of the people with diabetes were unaware about their condition. One out of every four adults aged between 20 and 59 years had diabetes and the frequency of this condition increased with age. Total prevalence of hypertension was 35.6% and four out of ten people with hypertension were unaware that they had this condition. Prevalence of hypercholesterolemia was 12.8% in men, 14.6% among women and 22.1% in older adults; 75.1% were unaware about having this condition.

Geographical distribution of overweight and obesity (Table 5): To analyze this information the country was divided arbitrarily in five regions: North (States of Baja California, Baja California Sur, Coahuila, Chihuahua, Durango, Nayarit, Nuevo Leon, Sinaloa, Sonora y Tamaulipas), Center (States of Aguascalientes, Colima, Guanajuato, Hidalgo, Jalisco, Estado de Mexico, Michoacán, Morelos, Puebla, Queretaro, San Luis Potosí, Tlaxcala, Veracruz y Zacatecas), South (States of Chiapas, Guerrero y Oaxaca), Southeast (States of Campeche, Quintana Roo, Tabasco and Yucatán) and Mexico City. The wealthiest states of the country are in the North region, whereas the states of the Central and Southeast regions and Mexico City, are a mix of low, middle and upper income; while the three states of the South Region are predominantly poor.

**Table 5:** Prevalence of overweight and obesity in each region of the country and age group

Condition/Age group	North	Center	South	Southwest	Mexico City	National
	**%**	**%**	**%**	**%**	**%**	**%**

Overweight						

Children	8.1	7.1	13.6	9.6	3.7	7.5

Adolescents	24.2	18.3	21.4	26.2	21.7	21.3

Women	40.6	36.1	40.3	36.4	34.5	37.1

Men	48.9	42.9	48.7	47.8	36.9	44.2

Older adults	44.2	41.8	41.0	43.3	43.2	42.7

Obesity						

Children	2.5	1.6	4.6	2.7	0.5	2.0

Adolescents	11.2	8.2	11.7	15.2	5.7	9.6

Women	29.4	23.6	27.6	32.8	16.1	25.0

Men	18.2	17.3	22.5	22.8	8.2	17.1

Older adults	31.4	25.3	30.5	32.1	21.0	27.2

Source: ENCOPREVENIMSS 2006.

Table 5 shows wide variations in the prevalence of overweight among regions and age groups. Overweight increases with age. The highest prevalence among children occurred in the Southeast region, while the North region had the highest prevalence for the other age groups. Obesity was more frequent in the South and Southeast regions.

Geographical prevalence of Type 2 diabetes mellitus and hypertension Table 6. The table shows the prevalence among individuals that were diagnosed previously and among those that were found as a result of the survey. For type 2 diabetes, the prevalence increases with age and the highest prevalence was found in the Southeast states. As for hypertension, the prevalence of this condition increases with age and an important proportion of individuals did not know about their condition. The highest prevalence was observed in women and older adults interviewed in the Northern states.

**Table 6:** Prevalence of Type 2 diabetes mellitus and hypertension among women, men and older adults in each region of the country

Condition/age group	Finding	North	Center	South	Southwest	Mexico City	National
		%	%	%	%	%	%
**Type 2 diabetes**							

Women	Previously diagnosed	7.9	6.2	6.5	10.6	7.2	7.4
	
	Finding in survey	1.8	1.8	1.2	1.9	1.1	1.7
	
	**Total**	**9.7**	**8.0**	**7.7**	**12.5**	**8.3**	**9.1**

Men	Previously diagnosed	6.5	6.4	7.5	10.6	6.9	7.2
	
	Finding in survey	1.1	1.2	2.0	0.7	1.2	1.1
	
	**Total**	**7.5**	**7.6**	**9.5**	**11.3**	**8.1**	**8.3**

Older adults	Previously diagnosed	30.7	29.5	25.7	32.1	30.4	30.0
	
	Finding in survey	1.3	1.6	2.5	1.3	1.9	1.6
	
	**Total**	**32.0**	**31.1**	**28.2**	**33.4**	**32.3**	**31.6**

**Hypertension**							

Women	Previously diagnosed	13.8	11.0	13.0	18.9	9.3	12.5
	
	Finding in survey	13.6	9.1	10.1	8.1	6.9	9.5
	
	**Total**	**27.4**	**20.1**	**23.1**	**27.0**	**16.2**	**22.0**

Men	Previously diagnosed	7.8	7.7	11.0	11.4	6.3	8.2
	
	Finding in survey	17.5	12.5	11.2	14.6	9.2	13.2
	
	**Total**	**25.3**	**20.2**	**22.2**	**26.0**	**15.5**	**21.4**

Older adults	Previously diagnosed	43.1	43.3	34.0	41.6	46.9	43.0
	
	Finding in survey	18.9	16.2	19.7	15.0	7.4	15.4
	
	Total	62.0	59.5	53.7	56.6	54.3	58.4

Source: Encoprevenimss 2006

## Discussion

PREVENIMSS' main goals were to increase coverage of preventive services based on health needs. The main findings of these surveys are the continuous increase of the coverage of preventive actions in the five age groups, and the ascertainment of the magnitude of old and emergent unmet needs among IMSS members, such as the high prevalence of anemia among children aged 0-4 years; the significant proportion of undiagnosed cases of hypertension in women, men and older adults and the proportion of people with overweight and obesity.

The coverage of preventive programs that were operational before the onset of PREVENIMSS was the highest since the first survey: Among children these were: measurement of height and weight, completed schemes of vaccination in children < 5 years old and congenital hypothyroidism detection in newborns. Preventive actions in women were, screening for cervical cancer; for women, men and older adults: type 2 diabetes and hypertension and pneumococcal vaccine for older adults.

Measuring coverage is particularly relevant to evaluate performance of individual programs within health systems and of individual countries regarding major international initiatives, such as the Millennium Development Goals (MDG). Mexico is on track to achieve the MDG-4 (two-thirds reduction between 1990 and 2015 in deaths of children under five years) [8] and the coverage of preventive actions that IMSS has achieved with its members is an underlying factor of these results, given the size of the population this institution covers.

A conceptual model has been proposed for assessing interventions to improve preventive services. This model comprises seven intervention components (reminders, feedback, education, financial incentives, regulatory interventions, organizational change and media campaign), four potential targets (patient, provider, organization and community) and key intervention features applicable to most of the intervention components (social influence, marketing, outreach, visual appeal, collaboration and teamwork, theory based, top management support and active learning strategies) [17].

We analyzed PREVENIMSS using this framework to identify its strengths and limitations. PREVENIMSS implemented several intervention components: reminders (through the booklets that address preventive activities), education (through educational activities aimed at promoting the use of preventive services); regulatory (through modifying the norms, regulations and criteria to provide preventive care); organizational change (through integrating all scattered preventive programs within a single strategy) and media campaign (through advertisements in radio, newspaper and television). The potential targets were, users, providers and the organization at central, district and local level. No financial incentives were considered as part of the intervention, neither actions promoting community participation were implemented as part of the program.

PREVENIMSS is the outcome of organizational changes that could be considered planned and developmental [18]. It was planned because it was deliberate, based on conscious reasoning and actions. It was developmental because it aimed at improving or correcting the processes to provide preventive care. Its design considered the demographic and epidemiological patterns of IMSS members and the IMSS' organizational strengths and weaknesses. This approach allowed defining the organizational changes that would contribute in assuring the implementation and sustainability of the program.

The finding of the high rate of anemia (19%) in children under four years of age, confirms what has been reported in other surveys carried out among the IMSS affiliated population (20.5%) [19]; the consequences of iron deficiency in the development of children have been widely described. This finding should be a wake-up call to analyze this situation in detail and to develop sound strategies aimed at tackling this problem. It is worth mentioning that IMSS members belong to the formal sector, which represents a regular income; thus, they are able to purchase food and commodities. It is also possible that certain socio-cultural factors like the dietary habits of children could have a negative influence on the possible impact of iron supplementation. Further studies are needed to address this topic.

Regarding the suffering from chronic conditions, ENCOPREVENIMSS reported that a significant proportion of interviewees were unaware of having either hypertension or diabetes. This suggests that PREVENIMSS must increase its screening activities in order to identify and diagnose cases for timely treatment. The interest in reinforcing preventive care for chronic diseases is due to its consequences for the individual and for the family, but also because these are high cost diseases that increase the burden for health care systems and for the society. Preventive services can contribute to avoid premature deaths and save resources.

The high rates of overweight, obesity, type 2 diabetes, and hypertension among interviewees mirrors what is observed in the actual provision of care; these are the main causes of visits to IMSS primary care facilities and among the chief causes of hospitalization. The growing burden of these conditions already represents a heavy toll for health systems [20].

The analysis of the geographical distribution of the prevalence of overweight, obesity, type 2 diabetes and hypertension showed important regional differences that allow making several assumptions. The percentage of individuals that did not know about their condition reflects an unmet need for preventive care. It is reasonable to assume a proportion of individuals without screening in a given year; however, given the magnitude of these conditions, this proportion should not be high. The analysis of the capacity to provide preventive care along with the knowledge about the actual demand and the information at local or regional level are necessary elements to estimate the actual and potential coverage and to set regional relevant goals for screening and to reinforce preventive actions in targeted age groups. Also, the geographical variations would indicate that certain socio-demographic conditions, such as income, access to food and lifestyle might have an important influence.

To interpret the findings of the surveys it is important to consider two main limitations in the design and focus of the evaluation. 1) The non-response rate as a source of bias. To compensate for the non-response rate we carried out two actions: a) To draw a larger sample size than needed (10%) and b) to replace the non-respondent households. However, the decision of replacing the household is a non-sampling error that carries out several potential problems, because the attempts to substitute non-responding households are time-consuming, prone to errors and a source of bias [21]. Given that the extent of non-response rate was below 10% in the four surveys, we may assume that this reduces the bias.

This strategy was focused to obtain an efficient sample design. The use of clusters allowed controlling costs and we aimed at maintaining the design effect as low as possible. It is well known that the default value for the design effect should be of 1.5 to 2.0, but this implied a considerable increase in the number of households and in the costs. We used a feasible number of clusters, within each, the smallest cluster size in terms of the number of households and this number was constant. It was also considered the information of previous surveys carried out in Mexico.

2) The surveys were not designed to measure or to evaluate the organizational change at the family medicine clinics. This shortcoming should be addressed in the short term and evaluating the organizational changes will provide key information to improve PREVENIMSS performance.

3) The survey did not collected information about the diphtheria-tetanus vaccine. This vaccine is routinely applied and the IMSS information system reports acceptable coverage figures however we should accept that this information should be included as part of the data.

Prevention is gaining attention in the international arena. In 2005, to address prevention and control of chronic diseases, the World Health Organization published a stepwise framework that comprises three core steps: 1. Estimate population needs and advocate for action, 2. Formulate and adopt policy, 3. Identify policy implementation steps. In a broader sense, IMSS actions that began in 2001 are similar to what WHO advices; PREVENIMSS identifies population needs and addresses the prevention component, while curative services, including primary care and hospital care are in charge of the control component. Theoretically, this is the right way. However, in a complex healthcare system, continuity and coordination of care between preventive and curative care, and among levels of care, requires strong advocacy and profound organizational changes [22].

The opportunity cost of preventive programs must be taken into account when designing health policies. Despite the potential benefits of preventive care, the fact is that most of primary care services are focused on providing curative care. Health policies in Mexico are oriented towards increasing coverage of health care and universal access. From our perspective, the focus should be to provide universal access to primary care services, which in turn comprises reinforcement of preventive services and provision of therapeutic care. The rational for this recommendation is straightforward: preventive care aims to avoid or delay the occurrence of diseases, to detect timely a disease, to avoid or delay complications when the condition is already present, to avoid premature deaths and to save resources. In fact, given that preventive care is appropriate for all, its provision is the first step to provide universal coverage, which in turn contributes in improving population health and reduces health disparities.

The rise in chronic diseases and the aging of the population are prompting decision makers and healthcare systems to look for prevention strategies that would help cope with this growing problem [23]. The impact of prevention services is not negligible; the financial resources saved can be used to pay for highly complex and more costly medical problems [24], yet this is an ongoing research field [25]. The resources allocated for preventive activities are far from enough and much more investment is needed. The aim is not to privilege preventive care over curative care, but to find the optimal balance between these forces while looking for cost-effective alternatives.

Analyzing the actual impact of PREVENIMSS on institutional performance is advisable. PREVENIMSS has already increased the demand for preventive care, which is due to both, changes in the organization that facilitated access to preventive services and users' demand as a result of the media campaigns and the information that was given personally when the users received the PREVENIMSS booklets. The increase in screening of diseases such as cancer, hypertension and diabetes will put further pressure on curative services to confirm the diagnosis and to provide timely and appropriate treatment to those already ill. This requires careful planning and reinforcement of current health services infrastructure to fulfill potential demand; currently there is no evidence of the impact of this increase on the actual provision of services.

Evaluating the impact of preventive actions would provide evidence of the cost-benefit of reinforcing prevention. To date there is no conclusive evidence of the benefit of preventive care for specific conditions. The analysis of the U.S. Preventive Service Task Force pointed out the lack of evidence on the health benefits of detecting type 2 diabetes, but it accepts that the benefits can be observable for hypertensives [26]. In fact the benefit of individual interventions, for example vaccines, screening of specific diseases or interventions aimed at improving lifestyle should be carefully analyzed from different perspectives. An adequate approach could be to measure effective coverage of preventive actions. Although, the significance as well as the difficulties and limitations in measuring effective coverage in Mexico have been addressed previously [27].

## Conclusion

After five years of its implementation, PREVENIMSS showed an important increase in coverage for the principal components of the program, and its working model could be applicable to reinforce nationwide preventive programs. The unsolved problems such as anemia, and the emerging ones such as overweight, obesity, among others, point out the need to strength preventive care through designing and implementing innovative programs aimed to attain effective coverage for those conditions in which prevention obtains substandard results

## Competing interests

The authors declare that they have no competing interests.

## Authors' contributions

GG, OM and SL contributed in developing the PREVENIMSS program; GG, HR, BA and SFC developed and conducted the surveys and carried out the statistical analysis of ENCOPREVENIMSS. RPC and HRM conceptualized and wrote the paper. All authors critically edited the manuscript, participated in the interpretation of data and read and approved the final version.

## Pre-publication history

The pre-publication history for this paper can be accessed here:

http://www.biomedcentral.com/1471-2458/10/417/prepub
